# Anterior chamber depth and angle-closure glaucoma after central retinal vein occlusion

**DOI:** 10.1186/s12886-016-0256-7

**Published:** 2016-05-31

**Authors:** Shiu-Chen Wu, Yung-Sung Lee, Wei-Chi Wu, Shirley H. L. Chang

**Affiliations:** Department of Ophthalmology, Chang Gung Memorial Hospital, 5 Fu-Hsin Rd., Kweishan, 333 Taoyuan Taiwan; Chang Gung University, College of Medicine, Taoyuan, Taiwan

**Keywords:** Angle-closure glaucoma, Anterior chamber depth, Neovascular glaucoma, Central retinal vein occlusion

## Abstract

**Background:**

The purpose of this study was to report the anterior chamber (AC) depth and the attack of angle-closure glaucoma (ACG) in eyes with the recent onset of central retinal vein occlusion (CRVO).

**Methods:**

This retrospective case series included 24 patients with recent onset of CRVO (within one month of attack) from July 2001 to December 2002. The mean follow-up period of the patients was 46 months (range: 3 to 92 months). AC depth was measured using an ultrasound biomicroscopy. Clinical data, including systemic disorders, intraocular pressure, and visual outcomes were recorded. The main outcome measures were AC depth in the diseased eye and the fellow eye of the same patient and the attack of ACG after CRVO.

**Results:**

The mean AC depth in the diseased eyes was significantly shallower than in the unaffected fellow eyes (2.43 ± 0.45 mm vs. 2.55 ± 0.46 mm; *p* < 0.001). Four patients (17 %) developed ACG after the onset of CRVO within one month of the CRVO attack. In these four patients, the mean AC depth in the diseased eyes was 1.91 ± 0.21 mm, which was much shallower than the eyes without ACG attack (2.53 ± 0.40 mm).

**Conclusions:**

AC depth is significantly shallower following the onset of CRVO. ACG can occur in patients after the onset of CRVO.

## Background

Glaucoma may be a contributory factor in the etiology of central retinal vein occlusion (CRVO), or may be a result of CRVO [1]. Neovascular glaucoma (NVG) is the most common form of glaucoma following CRVO [2, 3]. NVG mostly results from an elevated expression of vascular endothelial growth factor (VEGF) following the ischemic onset of vessel occlusion in the retina [4, 5]. However, shallowing of the anterior chamber (AC) following the onset of occlusion in CRVO, therefore resulting in asymmetric AC depths in the same patient, has been observed [6]. Clinical observations have shown that angle-closure glaucoma (ACG) can occur following CRVO without the formation of neovascularization in the angle of the eyes, although with a much lower incidence than NVG [7–11]. However, these previous studies are mainly case reports. The shallowing of AC is mainly observational without actual measurement of it. The purpose of this study was to investigate the AC depth by an ultrasound biomicroscopy (UBM) in patients with acute onset of CRVO and the incidence of ACG following CRVO in a consecutive series of CRVO patients. We hope that our findings can provide more in-depth observations and a better understanding of the association of CRVO and the onset of ACG.

## Methods

**Fig. 1:**
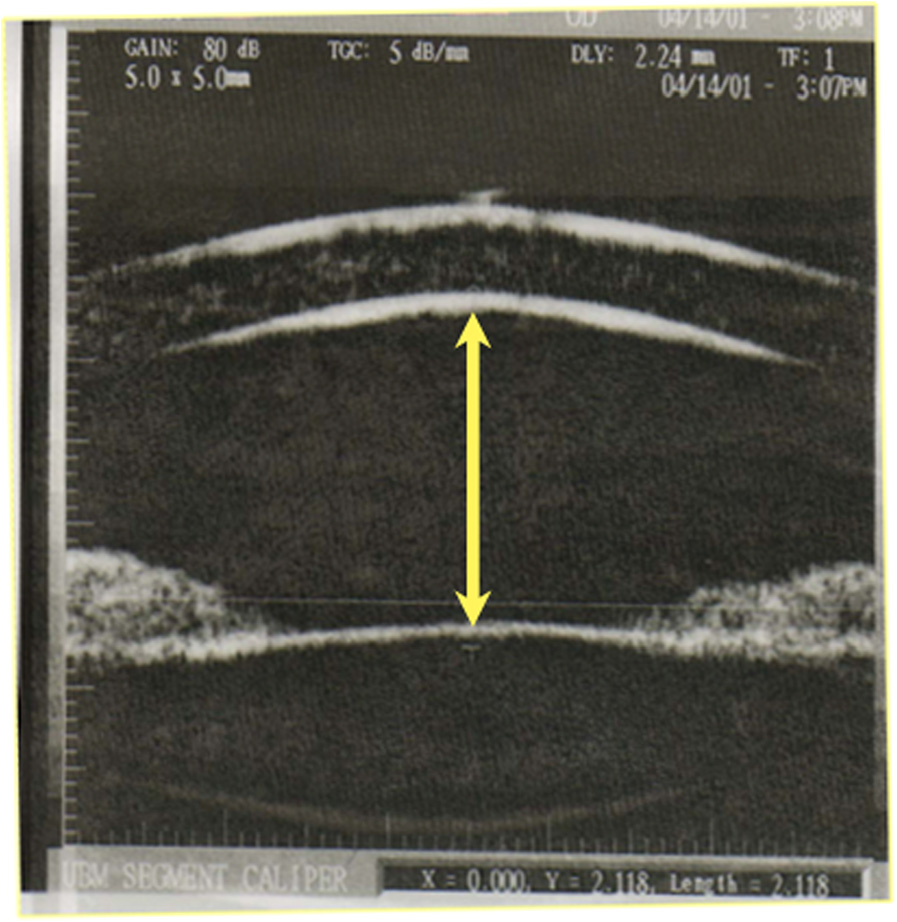
Anterior chamber depth was measured using an ultrasound biomicroscopy (UBM). Measurements were made from the anterior surface of the lens to corneal endothelium in the affected eye and fellow eye of the same patient (arrow)

This retrospective study was approved by the Institutional Review Board of Chang Gung Memorial Hospital and conforms to the provisions of the Declaration of Helsinki. Consecutive patients with recent onset of CRVO (within one month of attack) from July 2001 to December 2002 were collected and analyzed. Patients who had received panretinal photocoagulation before the measurement of AC depth were excluded from the study. Patients who had undergone cataract surgery before the onset of CRVO either in the diseased or fellow eyes were also excluded. AC depth was measured using an UBM (P40; Paradigm Medical Industries, Inc. Salt Lake City, UT). Measurements were made from the center of anterior surface of the lens to the central apex of corneal endothelium in the diseased eye and the fellow eye of the same patient (Fig. 1). Each eye was measured three times and the mean depth was calculated. Gonioscopy was also performed to evaluate the status of the angle in these patients. Clinical data, including systemic disorders, intraocular pressure, and visual outcomes, were recorded. All of the patients were followed-up for at least three months. All of the data were expressed as mean ± standard deviation (SD). The paired *t* test was used for comparisons of the AC depth between the diseased and unaffected eyes. The Shapiro-Wilk test was conducted to test the normality of the numerical variables. Statistical analysis was performed using SPSS (version 12.0 for Windows, SPSS Inc., Chicago, IL). A *p* value of less than 0.05 was considered to indicate statistical significance in this study.

## Results

Twenty-four patients (13 males, 11 females) were included in this study, with a mean age of 62.7 ± 12.3 years (range: 25–77 years). Hypertension was noted in five patients and diabetic mellitus was also noted in five patients. The affected left to right eye ratio was 12 to 12, and the ratio of ischemic CRVO to non-ischemic CRVO was 7 to 17. All of the diseased and fellow eyes were phakic. Twenty-three out of 24 (95 %) affected eyes of the 24 patients had a shallower AC depth than the unaffected eyes, with a mean AC depth of 2.43 ± 0.45 mm (range: 1.66–3.19 mm) in the diseased eyes and 2.55 ± 0.46 mm (range: 1.71–3.22 mm) in the unaffected eyes. The mean difference between the affected and unaffected eyes was 0.12 ± 0.10 mm (range: −0.03–0.34 mm) and was statistically significant (p <0.001; paired *t* test). The AC depths in the diseased and unaffected fellow eyes in the 24 patients with CRVO are shown in Table 1. Of the 23 patients who had a shallower AC depth of the affected eyes, we found that a difference of ≥0.1 mm between the affected and unaffected eyes accounted for 61 % of the patients (Table 2). Four patients (17 %) developed ACG within one month after the onset of CRVO occurred. The profiles of these four patients are shown in Table 3. Intraocular pressure elevation ranged from 28 to 37 mmHg. The angles in these four patients were closed without neovascularization. Of these four patients, two (50 %) had narrow angles and had received prophylactic laser iridotomy before the attack of CRVO. (case 1 and case 4 in Table 3) The other two patients (50 %) (case 2 and case 3 in Table 3) had very shallow AC without iris bombe following CRVO. Gonioscopy revealed a very narrow angle, only minimal anterior part of the trabecular meshwork being visible. Laser iridotomy was performed in these two patients. In these four patients, the mean AC depth in the diseased eyes was 1.91 ± 0.21 mm (range: 1.66–2.16 mm), and 1.99 ± 0.36 mm (range: 1.71–2.49 mm) in the unaffected eyes. The AC depth in these four eyes was much shallower than in the eyes without ACG attack (2.53 ± 0.40 mm) and the unaffected fellow eyes (2.66 ± 0.41 mm). In these four eyes, intraocular pressure (IOP) was under control after the application of laser iridotomy or anti-glaucoma medication. In the other 20 patients (83 %), IOP was within normal range (less than or equal to 22 mmHg) in both eyes. Gonioscopic evaluation showed open angles. Neither involved eyes nor un-involved eyes demonstrated neovascularization over the angles. Four eyes received pan-retinal photocoagulation following the onset of CRVO, and three eyes received vitrectomy and radial optic neurotomy for the ischemic CRVO. None of the eyes developed NVG after these interventions. The mean follow-up period of the patients was 46 months (range: 3 to 92 months).

**Table 1:** Anterior chamber depth in the diseased eyes with central retinal vein occlusion and the unaffected fellow eyes in 24 patients

AC depth	CRVO eyes	Fellow eyes	Difference^a^
Mean ± SD	2.43 ± 0.45 mm	2.55 ± 0.46 mm	0.12 ± 0.1 mm*
Range	1.66–3.19 mm	1.71–3.22 mm	−0.03–0.34 mm

*AC* anterior chamber, *CRVO* central retinal vein occlusion, *SD* standard deviation

^a^Of 24 patients enrolled, only one patient (4 %) had a deeper anterior chamber depth in the CRVO eye than the fellow eye

*p < 0.001 (Paired *t* test)

**Table 2:** Differences of anterior chamber depth in 23 patients with shallower anterior chamber depth in the central retinal vein occlusion eyes than the fellow unaffected eyes

Differences of AC (mm)	Number of eyes	Percentage (%)
0–0.05 mm	3	13
0.05–0.1 mm	6	26
0.1–0.2 mm	7	30
0.2–0.3 mm	5	22
>0.3 mm	2	9

*AC* anterior chamber

**Table 3:** Profiles of the patients with angle-closure glaucoma attacks after central retinal vein occlusion

Patient	Age	Laterality	ACD(mm)	ACF(mm)	DM	HTN	FU (m)
1	49	OS	1.650	1.709	No	No	93
2	56	OD	2.157	2.469	Yes	Yes	92
3	69	OS	1.975	2.002	No	No	92
4	61	OD	1.863	1.771	Yes	Yes	72

*ACD* depth of the anterior chamber of the diseased eyes, *ACF*, depth of the anterior chamber of the fellow eyes, *DM* diabetic mellitus, *F* female, *FU* follow-up, *HTN* hypertension, *M* male, *m* month, *OD* right eye, *OS* left eye

## Discussion

In this study, we found that the AC depth was significantly shallower in the eyes with CRVO attack than in the fellow eyes. Four patients (17 %) developed ACG after the onset of CRVO, and the mean AC depth in these eyes was only 1.91 ± 0.21 mm. Lin et al. [12] reported that in acute ACG patients in Taiwan, the mean AC depth (corneal thickness included) was 2.28 ± 0.23 mm. Subtracting the average corneal thickness of 0.5 mm, the AC depth was around 1.78 mm in the patients with ACG attack in Lin et al.’s study, and was consistent with our findings. An AC depth less than 2 mm may pose a greater risk for ACG clinically. ACG after CRVO has not been reported extensively. However, these issues are particularly important for Asians, as Asians have a shallower AC or a steeper cornea and a much higher prevalence of ACG than Caucasians [13–18].

Most of the previous studies on non-rubeotic ACG following CRVO are case reports or small case series and lack of actual measurement of AC depth [6–9]. Our study includes a series of CRVO patients with the actual measurement of AC depth. According to previous case reports, some common characteristics can be seen in those patients. First, temporary shallowing of the AC and reversible ACG without visible vascularization of the angle were noted. Second, neither evidence of pupillary block nor iris bombe was found; however, all of the patients had a rather forward displacement of the entire lens - iris diaphragm. Third, IOP control was usually achieved by medical treatment, and laser or surgical procedures were used as a back-up treatment if the IOP could not be controlled with anti-glaucoma medications. In our current study, we also had a very consistent clinical course. The four patients developing ACG had very shallow AC and revealed no vascularization over the angles. The mechanism of angle closure glaucoma was not due to papillary block in the two patients since they had received prophylactic laser iridotomy before. All the patients got good IOP control after laser surgery and glaucoma medications.

**Fig. 2:**
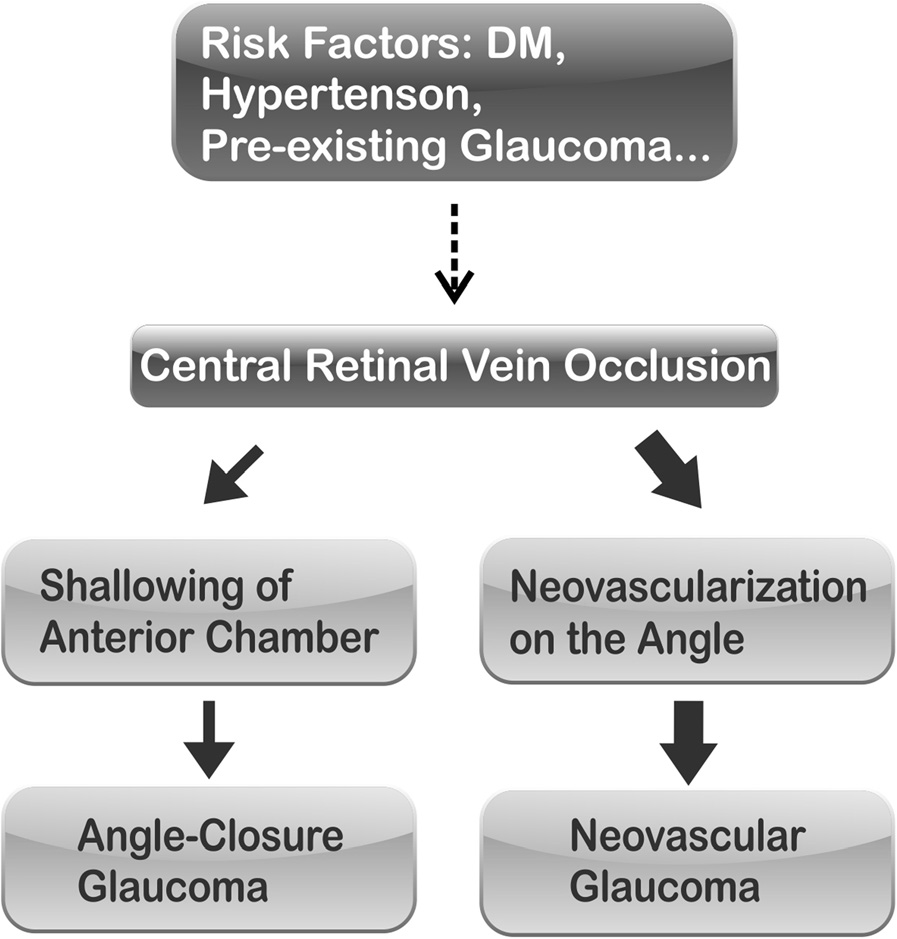
Scheme of central retinal vein occlusion (CRVO) and the association with glaucoma. Pre-existing glaucoma can be a risk factor and contribute to the onset of CRVO (broken arrow); after the attack of CRVO, and especially ischemic CRVO, patients develop neovascular glaucoma (NVG) at a higher frequency due to elevated vascular endothelial growth factor (VEGF) and formation of neovascularization in the angle of the eye (solid thick arrow). Or less often, patients develop angle-closure glaucoma (ACG) due to a shallow anterior chamber as demonstrated in this study (solid thin arrow)

The association of CRVO and glaucoma is complex. Pre-existing glaucoma is a well-characterized risk factor for CRVO [19–31]. CRVO occurs in 3.5 to 5 % of patients with primary open angle glaucoma [32]. Similarly, CRVO occurs in approximately 3 % of patients with ocular hypertension [32]. It is recommended that ocular hypertensive patients over the age of 65 years be treated to lower their intraocular pressure to below 25 mm Hg to reduce the incidence of CRVO [32]. Patients with CRVO have been found to have a higher IOP, and ocular hypertensive patients have an increased risk of developing CRVO [33, 34]. On the other hand, the majority of glaucoma attack after CRVO, and especially the ischemic type of CRVO, is NVG [2]. NVG usually occurs 90 days after CRVO, and therefore the term “90-day glaucoma” is used. Non-rubeotic ACG is much less common than the NVG, and usually occurs within one month of CRVO attack. A proposed scheme of central retinal vein occlusion (CRVO) and the association with glaucoma is shown in Fig. 2.

The mechanisms of AC shallowing after CRVO are uncertain. It has been hypothesized that marked swelling and vascular congestion of the ciliary body with anterolateral displacement after CRVO could lead to decreased AC depth. It is also likely that the increase in volume within the posterior segment, either due to blood or transudative fluid following CRVO pushes the lens-iris diaphragm forward. This condition may be under-diagnosed, especially in Asians with a shallower AC than Caucasians [13–18]. Further prospective studies including evaluation of AC depth, angle, and ciliary body changes in CRVO need to be performed to highlight this much neglected disorder.

The limitations of this study include its retrospective design, small sample size, and variable follow-up of the patients. In addition, no longitudinal follow-up of AC depth of these patients was performed. However, our comparative study showed significant shallowing of the AC following the onset of CRVO. This implies that the patients with recent-onset CRVO need to be monitored closely, not only for the presence of NVG, but also for attacks of ACG.

## Conclusions

AC shallowing by up to 0.3 mm in eyes after CRVO attack may occur compared with fellow unaffected eyes. It is important to evaluate AC depth and perform gonioscopic examinations in cases of recent-onset CRVO to assess the possible development of NVG as well as the frequently ignored ACG. An AC depth of less than 2 mm may pose a greater risk for ACG clinically. Secondary non-rubeotic ACG from CRVO must be distinguished from NVG, because the treatment and the prognosis may differ among the two disease entities.

## Abbreviations

AC, anterior chamber; ACG, angle-closure glaucoma; CRVO, central retinal vein occlusion; IOP, intraocular pressure; NVG, neovascular glaucoma; SD, standard deviation; UBM, ultrasound biomicroscopy; VEGF, vascular endothelial growth factor.
